# Accessing Lipophilicity and Biomimetic Chromatography Profile of Biologically Active Ingredients of Botanicals Used in the Treatment of Inflammatory Bowel Disease

**DOI:** 10.3390/ph15080965

**Published:** 2022-08-04

**Authors:** Mario-Livio Jeličić, Daniela Amidžić Klarić, Jelena Kovačić, Donatella Verbanac, Ana Mornar

**Affiliations:** 1 Department of Pharmaceutical Analysis, Faculty of Pharmacy and Biochemistry, University of Zagreb, A. Kovačića 1, 10000 Zagreb, Croatia; 2 Department of Medical Biochemistry and Hematology, Faculty of Pharmacy and Biochemistry, University of Zagreb, A. Kovačića 1, 10000 Zagreb, Croatia

**Keywords:** inflammatory bowel diseases, botanicals, biomimetic chromatography, shake-flask method, immobilized artificial membrane, plasma protein binding

## Abstract

In the present study, various procedures have been compared for the determination of lipophilicity, hydrophobicity, and plasma protein binding of curcuminoids, boswellic acids, andrographolides, and piperine as biologically active ingredients of botanicals used in IBD treatment. Our results have shown that IAM-HPLC assay is the most suitable one for lipophilicity determination of all analytes regardless of their class and botanical source. HSA-HPAC and AGP-HPAC assays revealed that all investigated compounds have a higher affinity for HSA which is the most abundant protein in human plasma. The high affinity of biologically active compounds to all biological structures (phospholipids and proteins) admonishes that their small portion is available for therapeutic effects in IBD patients. Our experimental research is complemented by various theoretical approaches based on different algorithms for pharmacokinetic properties prediction. The similarities between experimental and calculated values were evaluated using PCA and CA as a statistical tool. The statistical analysis implies that plasma protein binding is a complex process, and theoretical approaches still cannot fully replace experimental ones.

## 1. Introduction

**Figure 1:**
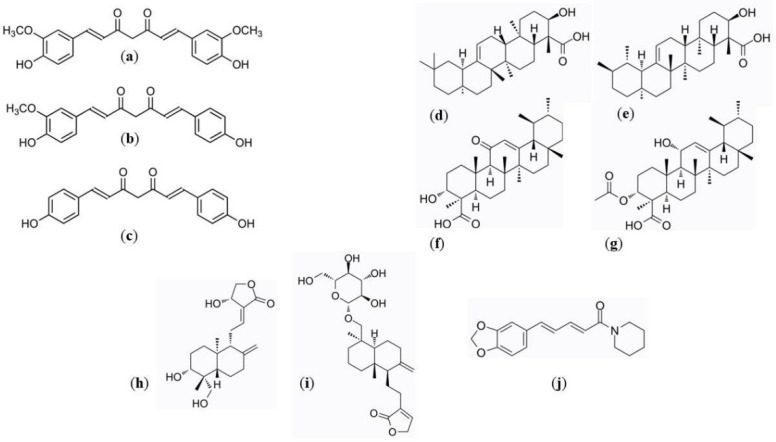
Chemical structures of curcuminoids (curcumin (**a**), demetoxycurcumin (**b**) and bisdemetoxycurcumin (**c**)), boswellic acids (α-boswellic acid (**d**), β-boswellic acid (**e**), 11-keto-β-boswellic acid (**f**) and 3-acetyl-11-keto-β-boswellic acid (**g**)), andrographolides (andrographolide (**h**) and neoandrographolide (**i**)) and piperine (**j**).

Inflammatory bowel disease (IBD), in its definition, includes two primary conditions —ulcerative colitis (UC) and Crohn’s disease (CD). These are chronic, relapsing gastrointestinal tract disorders that occur worldwide and affect people of all ages, with a high impact on their quality of life. More than 2 million Europeans and 1.5 million North Americans have IBD, and researchers report the rising incidence rates in newly industrialised countries in South America, Eastern Europe, Asia and Africa paralleled with the westernisation of diet and culture [1]. Proper IBD management requires early diagnosis, novel therapies, and management programs. Regardless recent improvements in IBD treatment, complementary and alternative therapeutic approaches for IBD have earned growing interest from patients, with a dynamic landscape of research in this area. According to Lin and Cheifetz [2], up to 60% of IBD patients use complementary and alternative medicine, with botanicals being the most popular. This growing popularity of botanicals is not surprising since patients perceive these products as safe and natural way of healing, free of side effects unlike conventional medicines. Amongst the botanicals, *Curcuma longa* L., *Zingiberaceae* (Turmeric), *Boswellia serrata Roxb. ex Colebr., Burseraceae* (Indian frankincense) and *Andrographis paniculata (Burm. f.) Wall. ex Nees, Acanthaceae* (Green chiretta) are the most popular herbal remedies used in IBD treatment. Despite the results of the relevant clinical studies showing the role of these botanicals in preventing and alleviating the symptoms of IBD, there is still considerable concern surrounding the bioavailability of their biologically active ingredients: curcuminoids as the most prominent compounds present in turmeric, boswellic acids as pentacyclic terpenoid molecules that make up 30% of frankincense resin and andrographolides as diterpene lactones (aglycone and glycosidic form) isolated from Green chiretta (Figure 1) [3,4,5,6]. Piperine is a piperidine alkaloid isolated from the fruits of *Piper nigrum* L., *Piperaceae* (Black pepper). Several studies unravel the therapeutic potential of piperine on amelioration of IBD as well as improvement in dissolution, stability of metabolic processes and membrane permeability of above mentioned biologically active compounds [7].

Numerous investigations have pointed out that research on herbal remedies, such as those mentioned above, has proved to be very difficult in all respects. Such a statement is attributed to their complex composition consisting of diverse chemical constituents each identical to a single active pharmaceutical ingredient of conventional medicine. However, a study providing deeper mechanistic insights into the bioavailability of biologically active ingredients of botanicals used in IBD treatment is essential to develop better treatment strategies in the future based on botanical products. The gold standard of bioavailability determination has long been considered a research study involving human volunteers. Albeit valuable, these studies are frequently demanding, unfeasible, subject to high overheads, and time consuming. In the light of these considerations, the scientific community’s attention towards alternative methods is increasing. Chromatographic methods are undoubtedly the most prominent ones since these methods using immobilized artificial membrane (IAM) provide the introduction to the separation mode of biological structures playing an essential role in drugs’ bioavailability, namely membrane phospholipids [8,9]. Likewise, high-performance affinity chromatography (HPAC) using human serum albumin (HSA) and α1-acid glycoprotein (AGP) as ligands within the chromatographic column has been successfully used to evaluate the interactions of drugs with serum proteins [10,11,12].

Unlike screening methodologies implemented on cell and animal models, these biomimetic chromatographic methods offer a superior reproducibility of the measurements, high-throughput data acquisition and environment-friendly, sustainable analytical approach [13]. Moreover, it is known that the biomimetic chromatography is based on physicochemical parameters, which allows elucidation of molecular mechanisms.

The main aim of this study was to assess the lipophilicity of biologically active ingredients of botanicals used in IBD treatment by miniaturized shake-flask procedure and evaluate their hydrophobicity, lipophilicity and affinity for plasma proteins using chromatographic techniques. Another goal of our research was to compare experimental indices with physicochemical parameters as well as adsorption and distribution properties estimated by artificial intelligence.

## 2. Results and Discussion

### 2.1. In Silico Calculation

Nowadays, computer-aided drug design is a crucial factor in the drug discovery and development process. As such, it was used as a starting point of our research to get some insight in physicochemical properties of biologically active ingredients of the most popular botanicals used in IBD treatment. The selection of parameters for in silico calculation was founded upon their relation to experimentally determined properties. 

Lipophilicity expressed as the logarithm of the partition coefficient between *n*-octanol and aqueous phase (log *P*) is one of the most important physicochemical parameters that has been found to affect a number of pharmacokinetic parameters. Accordingly, various software packages based on different algorithms are available for prediction of these properties. The log *P* values of biologically active ingredients of botanicals used in IBD treatment estimated by means of 15 theoretical approaches are summarized in Table 1. The highest values were obtained for lipophilic boswellic acids (average log *P* values were in the range from 5.56 to 6.57) followed by piperine (average log *P* value was 3.12) and curcuminoids (average log *P* values were in the range from 3.05 to 3.13). Generally, the lowest values were found for both compounds from the andrographolides group (average log *P* values were 2.06 and 2.11). The highest difference between log *P* values obtained by different theoretical approaches (up to high 4.42 units) found for boswellic acid reflects the incoherence of in silico approaches for highly lipophilic compounds. The lowest values for boswellic acids (up to 2.58 units lower than average calculated value) were obtained by iLOGP prediction that uses a rather new approach relaying on free energies of solvation in *n*-octanol/water and solvent accessible surface area for lipophilicity calculation. On the other hand, the highest values for this group of biologically active compounds (up to 1.86 units higher than average calculated value) were obtained using XLOGP3 software based on the well-known atomic method including corrective factors and knowledge-based library. For other less lipophilic compounds (curcuminoids, andrographolides and piperine), differences between calculated values were rather reasonable. Silicos-IT LogP software using an hybrid theoretical approach relaying on fragments and topological descriptors slightly overestimated log *P* values for all three curcuminoids (differences between calculated and average values were less than 0.91 units) while miLogP values calculated using group contribution model were lowest ones for both bicyclic diterpenoid lactones andrographolides (differences between calculated and average values were less than 1.00).

The solubility of biologically active ingredients of botanicals used in IBD treatment estimated by means of five theoretical approaches is presented in Table 1 and expressed as the logarithm of molar concentration (log *S*). As expected, the high lipophilicity of investigated compounds resulted in reduced solubility. The discrepancy between predicted log *S* values was acceptable for all compounds (Δ ≤ 1.74 units) except lipophilic boswellic acids (Δ was in the range from 2.61 units to 3.70 units) which confirms that highly lipophilic compounds are challenging for in silico approaches.

After the log *P* and log *S* value evaluation was completed, we have moved to the prediction of pharmacokinetic properties of target compounds using three platforms. We have used well recognized preADMET platform as well as two novel approaches to the prediction of pharmacokinetic properties pkCSM which relies on graph-based signatures as well as admetSAR platform based on models trained by state-of-the-art machine learning methods such as support vector machine, k-nearest neighbors, neural network etc. (Table 1).

All prediction procedures labelled boswellic acids as biologically active compounds with the high human intestinal absorption (obtained values were higher than 94%). Despite the widespread use of Indian frankincense, only few preliminary pharmacokinetic studies were conducted [14]. AdmetSAR web service offered us prediction not only of boswellic acids absorption but also their human oral bioavailability. This term is used to indicate the fraction of an orally administered dose that reaches the systemic circulation as intact drug, taking into account absorption, gastrointestinal stability and local metabolic degradation. Although the highest absorption among investigated biologically active compounds was predicted for boswellic acids at the same time their lowest bioavailability (from 50% to 53%) might be attributed to gastrointestinal instability of boswellic acids, high accumulation within the enterocytes, intestinal metabolism, extensive phase I metabolism observed for non-acetylated boswellic acids in human liver microsomes as well as saturable kinetics [15,16]. The most intriguing high absorption (obtained values were in the range from 82% to 98%) and bioavailability (obtained values were in the range from 60% to 64%) by these theoretical approaches was outlined for curcuminoids. Despite their demonstrated biological effects, the potential health benefits of curcuminoids are limited by their poor solubility, intestinal instability at a pH lower than 3 and higher than 6 and extensive first-pass intestinal and hepatic metabolism. As a result, different innovative strategies have been pursued to improve the absorption of curcuminoids including nanocrystals, emulsions, liposomes, etc. [14]. Amongst the investigated compounds the lowest human intestinal absorption (obtained values were between 62% and 81%) was predicted for diterpene neoandrographolide which can be related both to its pyranose ring and α,β-unsaturated lactone.

Regarding the plasma protein binding data, we found a discrepancy between values obtained by two tested theoretical approaches (Table 1). The recent studies confirmed the stable binding of biologically active ingredients of botanicals used in IBD treatment with plasma proteins and their complex formation supporting the data obtained by preADMET system [14,17]. Because boswellic acids represent lipophilic acids, it appeared reasonable to speculate that high plasma protein binding will be revealed. The predicted plasma protein binding of 100% for three boswellic acids needs to be interpreted with attention as such tight albumin-binding raises doubts regarding the efficiency of Indian frankincense in vivo and questions its pharmacological relevance.

Although diverse in silico values were obtained for biologically active ingredients of botanicals used in the treatment of IBD, this approach was our starting point in experimental design for lipophilicity, hydrophobicity and biomimetic chromatography profile assessment.

### 2.2. Shake-Flask Method

So far, the different approaches for the log *P* evaluation have been developed and described in literature [18]. We have chosen the reference shake-flask method on account of the fact that the gained results are scalable and easily transferable to other experimental and theoretical procedures. In our previous work [19] we have developed a miniaturized shake-flask methodology to increase the experimental throughput and to reduce the experimental effort and costs. Principles of green analytical chemistry were implemented in all analytical processes from sample preparation to their analysis. From the results presented in Table 1 it is evident that boswellic acids are highly lipophilic and such as that these compounds are not good candidate for lipophilicity determination by shake-flask methodology. Our preliminary investigation showed that shake-flask procedure failed for those compounds due to solubility issues even with the use of dimethylsulfoxide as a modifier. Therefore, at this point our investigation was directed to validation of procedure for curcuminoids, andrographolides and piperine according to ICH quidlines [20]. Results presented in Table 2 reveal linear (correlation coefficients (*r*) were higher than 0.999), sensitive (Limits of Quantitation (LOQ) were lower than 10 µg/mL), accurate (recoveries were within ±5%) and precise (Relative Standard Deviations (RSD) were lower than 3.61%) analytical methodology. The obtained log *D*_7.4_ values were in the range from 1.51 to 1.94 supporting the well-known applicability of the method for moderately lipophilic compounds (Table 3). Curcumin is a symmetric compound whose structure comprises three chemical units consisting of 2 aromatic ring systems containing *O*-methoxy phenolic groups linked through an α,β-unsaturated β-diketone moiety. Lower log *D*_7.4_ values obtained for demetoxycurcumin (Δ = 0.42 units) and bisdemetoxycurcumin (Δ = 0.22 units) are related with loss of one or both *O*-methoxy groups, respectively. On the other hand, pyranose ring in the structure of neoandrographolide slightly increased lipophilicity (Δ = 0.19 units) of this Green chiretta active ingredient compared to andrographolide. Moderate lipophilicity of curcumin and its novel synthetic structural analogues is observed in previous studies [21,22].

### 2.3. Chromatographic Methods

#### 2.3.1. Hydrophobicity Evaluation

In the course of the research, various stationary phases were used, differing in chemical structure and physicochemical properties. First, we have used C18 stationary phase which is alkyl-bonded phase modification of silica gel to which octadecyl carbon chains are attached. This stationary phase offers simple hydrophobic interaction with analyte and is available as thin-layer chromatography (TLC) plate and high performance liquid chromatography (HPLC) column. To get insight into hydrophobicity of investigated biologically active compounds, both solutions were used in this research.

In TLC measurements *R*_F_ value depends on the chemical structure of analyte and its interaction with stationary and mobile phase. All investigated compounds were strongly retained by hydrophobic stationary phase and for this reason mixtures of buffer and organic solvent were used as mobile phase. Methanol appeared to be the most suitable organic modifier for this purpose because it does not disturb the hydrogen-bonding network of water. As it was expected, with increase in the percentage of organic modifier in the mobile phase the increase in *R*_F_ values can be seen with accompanied decrease in *R*_M_ values expressed as *R*_M_ = log(1/*R*_F_ − 1). Measured *R*_F_ values were in the range from 0.1 to 0.7 obtained in a wide range of mobile phase organic modifier concentrations. Due to strong interaction with the stationary phase for hydrophobic analytes (11-keto-β-boswellic acid and 3-acetyl-11-keto-β-boswellic acid) this range was somewhat narrower, and mobile phases rich in an organic modifier had to be used. For the most hydrophobic analytes (α- and β-boswellic acids) no spot migration was observed regardless the percentage of the organic modifier in the mobile phase. The TLC hydrophobicity index, *R*_M0_, was determined by extrapolation of the organic modifier in the mobile phase to the zero concentration (Table 4) and regression coefficient for all analytes were higher than 0.979 with small values of standard error of the regression model (lower than 0.1041), which proves the high significance for hydrophobicity determination. Increasing concentration of the organic modifier in the mobile phase caused a slower decrease in the *R*_M_ values for less hydrophobic analytes than for more hydrophobic ones. Based on these results, generally, bicyclic diterpenoid lactones andrographolides show the least hydrophobic character followed by piperine and curcuminoids while extremely high was found for boswellic acids. Increase in the hydrophobicity within curcuminoids and andrographolides is related to increase in lipophilicity determined by shake-flask method (Table 3). 

Functional groups present in the structure of these compounds increase their hydrophobicity and lipophilicity in the same manner. The presence of keto group in the structure of boswellic acids was crucial for applicability of TLC procedure in determination of hydrophobicity of these compounds. As it was expected, the acetyl group present in the structure of 3-acetyl-11-keto-β-boswellic acid increased hydrophobicity of this boswellic acid compared to 11-keto-β-boswellic acid (Δ = 1.53 units).

Hydrophobicity of analytes was also investigated using high-performance liquid chromatography and C18 stationary phase. The analytical approach to both indirect chromatographic determination of hydrophobicity was coherent, still each technique provided advantages and limitations. The adaptability of TLC allowed us the analysis of all investigated compounds simultaneously, while the most important practical advantage of HPLC was process automatization and online detection. 

The principles of hydrophobicity determination by HPLC are characterized by the logarithm of retention factor (*k*), defined as log*k* = log (*t*_R_−*t*_0_)/*t*_0_, where *t*_R_ is retention time of analyte and *t*_0_ is the dead time. The direct measurement of log*k* in buffer of investigated biologically active compounds was impossible due to their high hydrophobicity which led to very long retention time and at the same time to extensive broadening of the peaks. The percentage of methanol in the mobile phase was optimized so that the analytes had retention time between dead time and a maximum of 20 min keeping the analysis time as short as possible, and the peak shape acceptable (peak asymmetry factor were between 0.8 and 1.2). By evaluating the profiles of log*k* values for all methanol fractions, the regular changes in retention with increasing methanol ratios were observed. The HPLC hydrophobicity index, log*k*_0_, was determined by extrapolation of the organic modifier in the mobile phase to its zero concentration. The results of linear regression are listed in Table 4. For all analytes, high values of regression coefficients (higher than 0.970) and small values of standard error of the regression model (lower than 0.0581) were achieved. The interaction of analytes with stationary phase was consistent within both C18 chromatographic systems. Still, except in the case of neoandrographolide the obtained HPLC hydrophobicity indices were slightly lower than those obtained by C18-TLC assay (from 0.18 to 1.07 units). Although a good chromatographical separation of curcuminoids and piperine in different mobile phases was observed, this method failed in discriminating log*k*_0_ values of curcuminoids and piperine (Δ = 0.01 units). As it can be seen in the case of the highly hydrophobic substances boswellic acids, these compounds are beyond the reach of C18-HPLC assay. Their retention time using mobile phase with high 80% of methanol was over 17 min. The polarization of the stationary phase in the presence of increasing content of methanol as well as increased hydrophobic interactions of analytes with stationary phase explains the disruption of the linearity of the regression and unsuitability of C18-HPLC system for such highly hydrophobic compounds.

#### 2.3.2. Lipophilicity Evaluation

Both C18-TLC and C18-HPLC assays gave us insight into hydrophobicity of investigated compounds. Although these results are valuable, to evaluate bioavailability of biologically active compounds more data should be collected and discussed. IAM columns provide potential to simulate membrane permeability since the amphiphilic character of phospholipid functional groups plays an important role in IAM retention especially when charged molecules are analysed. In order to clarify similarities and dissimilarities between chromatographic approaches the analytical procedure used for determination of HPLC lipophilicity index, log*k*_0 IAM_, was comparable with above-described procedure for C18-HPLC assay. 

Our findings would seem to imply that IAM-HPLC assay is the most suitable one for determination of lipophilicity of all analytes regardless of structure and botanical source (Table 4). In addition to the wide applicability of the method, the high values of correlation coefficients (higher than 0.980) and the small values of standard error of the regression model (lower than 0.0872) support the idea that IAM-HPLC assay would lend itself well for use in membrane permeability studies. The all investigated compounds retained less on IAM stationary phase than on C18 one, still log*k*_0 IAM_ values obtained for all analytes except piperine (Δ = 0.01 units) are generally higher than log*k*_0_ values (differences were in the range between 0.37 to 1.22 units). In the case of boswellic acids, the position of two methyl groups on C-19/C-20 (α-type: germinal groups on C-20; β-type: vicinal groups on C-19/C-20) had an influence on interaction with phospholipids. The oleanane (β) type interacted strongly with phospholipids compared to ursane (α) type. The pharmacologically interesting 11-keto-β-boswellic acid had a lower affinity compared to β-boswellic acid (Δ = 2.60 units), most likely due to carbonyl group at C-11 of pentacyclic triterpene. Conversely, the acetyl function at C-3 position increased log*k*_0 IAM_ for more than one unit. The evidence from this study points toward the idea that IAM stationary phase may be useful for the evaluation of lipophilicity of highly lipophilic compounds, such as boswellic acids, which distinguish this experimental approach among the others. On the other hand, low solubility and high affinity of boswellic acids for phospholipids leads to question of their bioavailability and pharmacological relevance. Consequently, future work in the field of pharmaceutical development needs to be performed to get the most of Boswellia serrata extract as it was assigned orphan drug status in 2002 by the European Medicine Agency for treatment of peritumoral edema [23]. All analytes from curcuminoid group compared to boswellic acids showed to some extent lower affinity to phospholipid. The less retained curcuminoid on IAM stationary phase was curcumin whose structure comprises of the two phenolic-methoxy groups in the opposite sides of curcumin backbone. The IAM assay revealed the increase in log *k*_0 IAM_ values for each methoxy function attached to phenolic backbone from 0.1 to 0.2 units. Still, as in the case of Boswellia serrata extract an innovative improvement in pharmaceutical development of Curcuma extract is considered a very reasonable approach for improving their bioavailability [24]. Moderate affinity of andrographolides to IAM stationary phase among investigated compounds is in accordance with their lower retention in C18 stationary phase. Still, the presence of sugar residue in the structure of labdane glucoside diterpene neoandrographolide considerably affected its affinity to phospholipids (difference in log*k*_0 IAM_ values was 1.18 units) than alkyl chains (difference in log*k*_0_ values was 0.95 units and *R*_M0_ values 0.08 units). Before interpreting results for piperine, it should be stated that the IAM stationary phase surface is mainly zwitterionic at pH 7.4. The positively charged choline moieties are located in the outer part of the IAM layer. By way of contrast, the negatively charged phosphate groups are present in the phase’s inner part. This would appear to indicate that retention of piperidine alkaloid piperine in IAM stationary phase is mostly due to interaction of its positively charged nitrogen atom and phosphate groups more in-depth of stationary phase.

#### 2.3.3. Plasma Protein Affinity Evaluation

In the consecutive set of studies, plasma protein binding of biologically active compounds was evaluated by bioaffinity chromatography using both assays HSA-HPAC and AGP-HPAC, respectively. The selection of HSA-HPAC assay for protein binding assessment purposes was substantiated by the fact that HSA is the most abundant protein found in human plasma (around 60%), and accordingly the most emerged versatile carrier for therapeutic agents. Albeit the AGP concentration in the plasma ranges up to only 3%, its concentration depends on the disease state and can increase significantly in patients with IBD. Therefore, our study has gone some way towards enhancing our understanding of plasma protein binding of selected biologically active compounds particularly in patients with IBD [25].

The gradient retention times obtained by HSA-HPAC and AGP-HPAC assays were standardized using a calibration set of compounds with known percentage of plasma protein binding data and procedure described in our previously published study [12]. Although the utmost care was put in preserving the performance of both biomimetic columns over the time verification of protein stationary phase was essential and was ensured by analysis of racemic mixture of warfarin during six consecutive days that revealed the repeatable separation of enantiomers (average resolution factors between enantiomers were 2.27 with RSD value 1.93% for HSA while 1.63 with RSD value 1.04% for AGP) (Table S1). This verification assured that the protein affinity indices obtained by HPAC assays depict not only unspecific generally lipophilicity-driven interactions, but also highly specific recognition forces responsible of enantioselectivity.

Table 5 highlights that HSA strongly attracted biologically active ingredients of botanicals used in IBD treatment. Beside andrographolides, all investigated compounds have exceptional affinity to HSA (more than 95%). The high affinity for protein molecule possibly relies on the hydrophobic regions of compounds (aliphatic and aromatic rings) that improved their ability to penetrate the hydrophobic cavity. Hydrogen bonds might also be involved in the sharing of hydroxylic or phenolic protons with numerous amide carbonyl moieties of HSA [17,26].

We have found that our analytes had generally lower affinity for AGP than HSA (AGP binding proportions were from 10% to 42% lower than values obtained for HSA) (Table 5). The differences were specific for each botanical group. The lowest deviations were obtained for highly lipophilic boswellic acids (differences were around 10%), a difference of 15% was obtained for all curcuminoids, while the least lipophilic andrographolides and piperine had the highest differences between HSA and AGP binding proportions from 23% to 43% (neoandrographolide, the only compound in glycosidic form).

High affinity to phospholipids demonstrated by IAM-HPLC assay supported with reversed but still noteworthy inactivation by protein binding presented by both assays reinforces the claim that a small portion of biologically active ingredients of botanicals used in IBD treatment are available for therapeutic effects [14]. Moreover, the most lipophilic compounds and consequently ones with the most compromising bioavailability have the highest affinity for both plasma proteins which makes them therapeutically inactive.

### 2.4. Comparison of Computational and Experimentally Observed Values Using Statistical Methods

**Figure 2:**
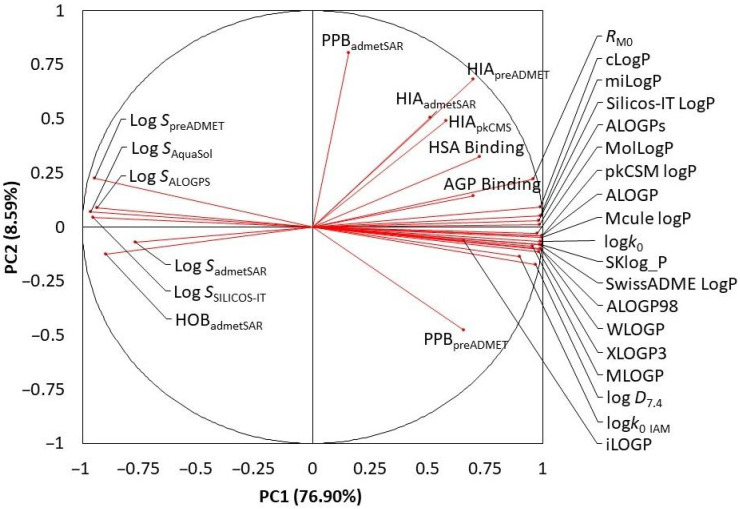
Results of PCA analysis reflecting correlation between predicted and experimentally observed values.

To evaluate the similarities between calculated and experimentally observed values, and their mutual correlation, statistical tools such as Principal Component Analysis (PCA) and Cluster Analysis (CA) were used. Obtained results of PCA showed that first two principal components describe 85.49% of data variability, where first component (PC1) and second (PC2) describe 76.90% and 8.59% of data variability, respectively (Figure 2).

Looking at the bi-plot we can see that most of the data is densely placed far right on the PC1 axis, mostly comprised of predicted log *P* values, as well as experimentally observed log *D*_7.4_ values and hydrophobicity and lipophilicity indices obtained using TLC and HPLC techniques. Similar orientation of these vectors, with small angles between them and similar PC1 values mean that predicted values are relatively close to those experimentally observed, which implies that used calculation tools have good prediction capability.

The second cluster formed is mostly comprised of predicted log *S* values representing the predicted solubility/hydrophilicity of examined biologically active compounds. Seeing that they are placed opposite of predicted and experimentally observed lipophilicity values says that there is a negative correlation between them, which is in line with our expectations. The only one pharmacokinetic parameter that correlates with predicted log *S* values is human oral bioavailability observed by the admetSAR software package. As mentioned above, the bioavailability of xenobiotics is a complex process. According to our results the solubility of investigated biologically active ingredients of herbals used in IBD treatment plays a crucial role in their oral bioavailability.

The third cluster is formed from human intestinal absorption values obtained by different prediction procedures. These values are closed to various hydrophobicity and lipophilicity parameters indicating acceptable relationship.

The last cluster, rather dispersed, is comprised of predicted plasma protein binding values accompanied by experimentally observed affinity to AGP and HSA protein. However, predicted plasma protein binding values are poorly corelated with experimentally observed ones which implies that prediction of affinity of our biologically active compounds to both plasma proteins is rather complex.

**Figure 3:**
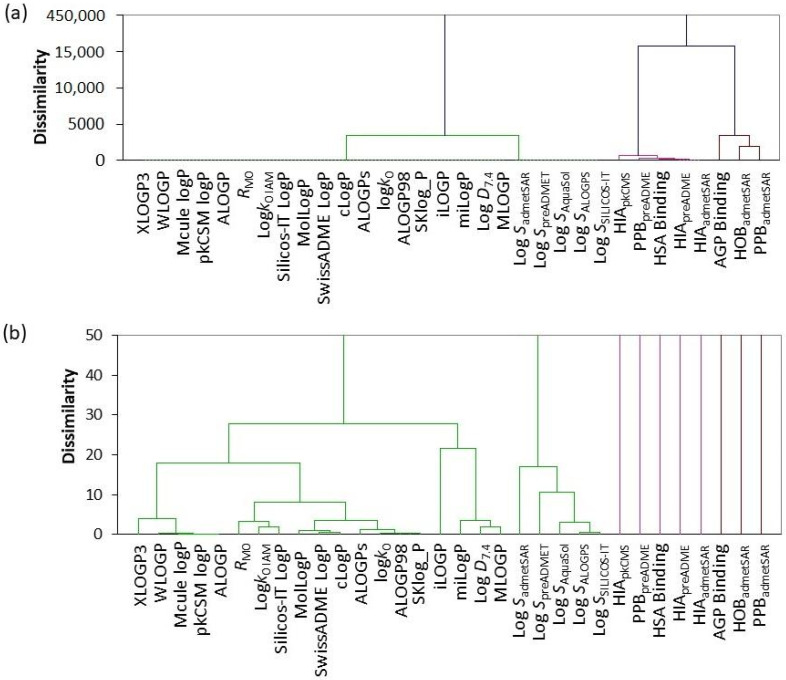
Results of CA showing (**a**) observed dendrogram in full scale from 0–450,000 with brake after 15,000, and (**b**) dendrogram showing only 0–50 range of dissimilarity values.

On the other hand, Figure 3 highlights the results of CA that reflect the similarity of observed values. As expected, the formation of two main clusters which are dissimilar one to another, one comprised of data related to lipophilicity and solubility and other representing the data related to plasma protein binding, human intestinal absorption and human oral bioavailability of biologically active ingredients of botanicals is evident (Figure 3a).

For the better visualisation of CA results, due to the high dissimilarity of those two clusters, Figure 3b shows a fraction of the main dendrogram. We can see that the green cluster is comprised of two subclusters. The first subcluster shows results related to lipophilicity. Experimentally obtained log *D*_7.4_ values are closest to values predicted by the MLOGP procedure, followed by procedure of Molinspiration Cheminformatics Group. Log *P* values predicted by ALOGP98 and SKlog_P are closest to those experimentally observed by C18-HPLC assay, followed by predicted ALOGPs values. With best accuracy Silicos-IT LogP values predict log*k*_0 IAM_ ones collected using IAM-HPLC assay. Following the dendrogram we can notice which model predicted log *P* values with the best accuracy, leaving iLOGP with the highest dissimilarity implying that it showed poor performance. Second subcluster is comprised of log *S* values showing the order of similarity between used prediction tools.

The second main cluster, related to plasma protein bindings, shows that the predicted values are not as accurate as ones observed experimentally, which was also observed in PCA.

## 3. Materials and Methods

### 3.1. Chemicals

Analytical grade standards of biologically active ingredients of botanicals (curcumin, demetoxycurcumin, bisdemetoxycurcumin, α-boswellic acid, β-boswellic acid, 11-keto-β-boswellic acid, 3-acetyl-11-keto-β-boswellic acid, neoandrographolide and piperine) were purchased from Sigma-Aldrich (St. Louis, MO, USA), while andrographolide (≥98.0%) was obtained from TCI (Tokyo, Japan). Warfarin (PESTANAL^®^, analytical standard) was obtained by Sigma-Aldrich. Buffer solutions were prepared using phosphate buffer saline (PBS) tablets (Sigma Aldrich), di-sodium hydrogen phosphate dihidrate (buffer substance for chromatography) and sodium dihydrogen phosphate dihydrate (EMSURE^®^ reagent Ph. Eur.) both by Merck KGaA, Darmstadt, Germany. Organic solvents *n*-octanol (gradient grade for liquid chromatography, ≥99%) and methanol (gradient grade for liquid chromatography LiChrosolv^®^ Reag. Ph Eur.) as well as formic acid (for LC-MS, LiChropur^®^, 97.5–98.5%) and dimethyl sulfoxide (suitable for HPLC, ≥99.7%) were obtained by Merck KGaA. Dead volume of HPLC system was evaluated using sodium nitrate (reagent for USP/NF monographs) by J.T. Baker, Gliwice, Poland. Ultrapure water was produced using an Ultra Clear UV water purifying system (SG Water, Barsbuttel, Germany); resistivity > 18 MΩ/cm at 25 °C and total organic carbon < 5 ppb. 

### 3.2. Methods

#### 3.2.1. In Silico Calculation

Several software packages were used for lipophilicity calculations, whereas each is based on different algorithms. Six different log *P* values (iLOGP, XLOGP3, WLOGP, MLOGP, Silicos-IT LogP, SwissADME LogP) were attained using a web service developed by the Molecular Modeling Group of the Swiss Institute of Bioinformatic (available online: http://www.swissadme.ch, accessed on 1 June 2022). ALOGPs values were calculated using a software developed by Virtual Computational Chemistry Laboratory (available online: http://www.vcclab.org, accessed on 1 June 2022). Free web property calculation service developed and maintained by Molinspiration Cheminformatics Group was used for calculation of miLogP values (available online: https://www.molinspiration.com, accessed on 1 June 2022), while Mcule logP values were derived using an online drug discovery platform developed by Mcule, Inc. (available online: https://mcule.com/apps/property-calculator/, accessed on 1 June 2022). OSIRIS Property Explorer as Integral part of Actelion’s system (available online: https://www.organic-chemistry.org/prog/peo/, accessed on 1 June 2022) calculated cLogP values. Free on-line software tool (available online: https://molsoft.com/mprop/, accessed on 1 June 2022) from Molsoft L.L.C. was used for acquiring MolLogP values. In the same vein, the web-based application for drug-likeness prediction preADMET QSARhub available online: https://preadmet.qsarhub.com/druglikeness/ (accessed on 1 June 2022) was used for calculation of ALOGP98 values.

Software packages by the Molecular Modeling Group of the Swiss Institute of Bioinformatic and Virtual Computational Chemistry Laboratory enabled solubility prediction (log *S*_SILICOS-IT_ and log *S*_ALOGPS_). This parameter as AquaSol was also available at ChemDB Portal available at https://re.edugen.wiley.com/, accessed on 1 June 2022.

The pkCSM integrated freely available web server platforms for predicting small molecule pharmacokinetic properties from the University of Melbourne and University of Cambridge available at: http://biosig.unimelb.edu.au/pkcsm/, accessed on 1 June 2022 was used for calculation of log *P* values and pharmacokinetic property (Human Intestinal Absorption). Web service preADMET, available online at https://preadmet.webservice.bmdrc.org/adme/, accessed on 1 June 2022 from Yonsei University, was used to predict lipophilicity (SKlog_P), solubility (log *S*_preADMET_) and biological properties (Human Intestinal Absorption and Plasma Protein Binding). Finally, an upgraded version of the comprehensive source and free tool for evaluating ALOGP values, solubility (log *S*_admetSAR_) and similar chemical pharmacokinetic properties (Human Intestinal Absorption, Human Oral Bioavailability and Plasma Protein Binding) admetSAR is available online: http://lmmd.ecust.edu.cn/admetsar2/, accessed on 1 June 2022.

#### 3.2.2. Shake-Flask Method with Chromatographic Analysis

##### Sample Preparation

Stock solution of biologically active ingredients of botanicals used in IBD treatment were prepared using dimethylsulfoxide in concentration of 2 mg/mL. Working solutions were prepared daily by dilution of the stock solutions with PBS (20 mM, pH 7.4) pre-saturated with *n*-octanol. Before the injection into the HPLC system all solutions were sonicated for 15 min at room temperature in Elmasonic XtraTT (Elma Schmidbauer, Singen, Germany) and filtered through 0.2 μm polyethersulfone filters (Obrnuta faza, Pazin, Croatia). 

The pre-saturated solutions of *n*-octanol and PBS (20 mM, pH 7.4) were used for determination of logarithm of distribution coefficient (log *D*_7.4_). The *n*-octanol and aqueous phase (mixture 1:1, *v*/*v*) were mutually saturated for 24 h in an orbital shaker-incubator ES-20/60 (Biosan, Riga, Latvia) at 100 oscillations per min at 25.0 ± 0.1 °C. The prepared mixture was left resting for at least 12 h in the thermostat at 25.0 ± 0.1 °C to ensure complete separation of the two phases. Until analysis, separated saturated solutions were kept in refrigerator at 4 °C.

Shake-flask procedure was conducted in amber 2 mL HPLC vials. 300 μL of the working solution was added to 1500 μL of *n*-octanol pre-saturated with PBS. To prevent material loss due to volatilisation, the two-phase system nearly filled the entire volume of the test vessels. All *n*-octanol–buffer mixtures were first vortexed for 1 min and then shaken for 1 h (150 oscillations per min) at room temperature (25.0 ± 0.1 °C) to reach equilibrium and phase distribution. Afterward, the samples were centrifuged using a Z 326 K tabletop centrifuge (HERMLE Labortechnik, Wehingen, Germany) for 30 min at 4000× *g* and 25 °C to ensure that possible emulsions were removed.

##### Sample Analysis

After equilibration and phase separation, the aqueous phase of all samples was analysed on an Agilent HPLC 1100 (Agilent Technologies, Waldbronn, Germany) with diode array detection. Analysis was conducted with a Zorbax SB C18 column (length 150 mm, i.d. 4.6 mm, particle size 5 μm) by Agilent Technologies. 10 min chromatographic runs were performed at 25.0 ± 0.1 °C in isocratic mode using a methanol/water mixture (60/40, *v*/*v*) with addition of 0.1% of formic acid as mobile phase modifier at a flow rate of 1.0 mL/min. The mobile phase was filtered through a membrane filter, no. 66, diameter 47 mm, pore size 0.45 µm (Supelco, Bellefonte, PA, USA). The vials were placed in the rack on the autosampler at 25 °C. The injection volume was 10 µL, with a needle offset of 0.5 mm. To avoid any carryover of the analyte-containing *n*-octanol phase, the outside of the syringe was subsequently washed in methanol before injection. The absorbance of the analytes during a chromatographic run was collected in the spectral range 200–400 nm, and the detection wavelength for each analyte was the one providing the maximum peak height.

#### 3.2.3. Chromatographic Methods

##### Sample Preparation

Stock solutions of analytes and calibration standards were prepared by dissolving 10 mg of each solute in 10 mL of methanol and kept at 4 °C. Working solutions were freshly prepared at the beginning of each day by dilution of the stock solutions to 100 μg/mL with methanol for TLC measurements or mobile phase for HPLC measurements. Before use, the samples were sonicated (Elmasonic XtraTT, Elma Schmidbauer) for 10 min at 25 °C and all working solutions were filtered through 0.2 µm polyethersulfone filters (Obrnuta faza). To protect analytes from photodegradation, the amber glass HPLC vials were used.

##### C18-TLC Assay

Analyses were carried out using a commercially available 10 cm x 20 cm RP-18 TLC plates with fluorescent indicator (Merck KGaA). 5 μL of each working solution (100 μL/mL) was applied to the plates as 5 mm bends, 10 mm from the lower edge and 15 mm from sides of plates. The mobile phases were prepared by mixing the respective amounts of PBS (20 mM, pH 7.4) and methanol from 50% to 80% of organic solvent in 5% increment. The vertical flat-bottom chamber (Camag, Muttenz, Switzerland) with a stainless-steel lid was saturated with mobile phase for 30 min. The plates were developed to a distance of 9 cm at room temperature, dried in the open air for 5 min and visualized under λ = 254 nm UV light (UV Cabinet with Dual Wavelength UV lamp by Camag).

##### C18- and IAM-HPLC Assays

C18-HPLC assay was carried out at Agilent HPLC 1100 and Zorbax SB C18 chromatographic column (length 250 mm.6 mm, particle size 5 µm) by Agilent Technologies, while IAM-HPLC assay was conducted using IAM P.C.DD2 chromatographic column (length 50 mm, i.d. 3.0 mm, particle size 300 Å) by Regis Technologies (Morton Grove, IL, USA). Retention data were collected at 25.0 ± 0.1 °C using isocratic method working with mobile phases, mixing the respective amounts of PBS (10 mM, pH 7.4) and methanol from 35% to 80% of organic solvent in 5% increment. Each mobile phase was shaken vigorously and filtrated through membrane filter, no. 66, diameter 47 mm, pore size 0.45 µm by Supelco and degassed by sonication 5 min before use. The injection volume was set at 10 µL, and the measurements were carried out at flow rate 1.0 mL/min. Natrium nitrate solution (0.1 mg/mL in mobile phase) was used as the marker of dead-time. The absorbance of the analytes during a chromatographic run was collected in the spectral range of 200–400 nm, and the detection wavelength for each analyte was the one providing the maximum peak height.

##### HSA- and AGP-HPAC Assays

The binding of biologically active compounds to the plasma proteins was investigated using affinity chromatographic columns by ChromTech (Cedex, France) containing immobilized human serum albumin (HSA) (Chiralpak-HSA, length 50 mm, i.d. 4.6 mm, particle size 5 µm) and α1-acid glycoprotein (AGP) (Chiralpak-AGP, length 50 mm, i.d. 4.6 mm, particle size 5 µm) and Agilent HPLC 1100 instrument. The 20 mM potassium phosphate buffer with the pH adjusted to 7.4 and iso-propanol were used as mobile phase components A and B. Retention data were collected at 25.0 ± 0.1 °C using gradient program as follows: 0 min/0% B, 6 min/30% B, 15 min/30% B and 20 min/0% B. Injection volume was set at 10 µL and the measurements were carried out at 25.0 ± 0.1 °C with flow rate of 1.5 mL/min. The absorbance of the analytes during a chromatographic run was collected in the spectral range 200–400 nm, and the detection wavelength for each analyte was the one providing the maximum peak height.

#### 3.2.4. Statistical Analysis

Statistical analyses for shake-flask method and biomimetic chromatography were conducted using Microsoft Excel v16.0.14026.20270 (Microsoft Corporation, Redmond, WA, USA).

PCA and CA analysis was performed using XLSTAT by Addinsoft (Paris, France) with the aim of correlation and better visualization of experimental and calculated data. A matrix 30 × 10 was created where number of rows represented data related to each analyte and columns represented data obtained from each prediction tool. Statistical data processing was performed using the principal component analysis with Pearson’s correlation and cluster analysis using Euclidean distance measures and Ward’s agglomerative clustering.

## 4. Conclusions

This research describes the shake-flask and chromatographic investigations used to understand the interactions of bioavailability challenged biologically active ingredients of botanicals used in IBD treatment with cell membrane and plasma protein models.

Our findings imply that IAM-HPLC assay is recommended as the most suitable method for determining the lipophilicity of these classes of biologically active compounds, regardless of their structure and botanical source. Moreover, the excellent linearity of the regression models supports the idea that IAM-HPLC assay is useful in membrane permeability studies. The standardized HSA- and AGP-HPAC assays were successfully applied to evaluate of plasma protein binding of selected biologically active compounds. Our results revealed that investigated compounds have a higher affinity for HSA than AGP. A high affinity to phospholipids supported with meaningful inactivation by plasma proteins presented by both assays underpins the claim that a small portion of biologically active ingredients of botanicals used in IBD treatment are available for therapeutic effects.

Our research was empowered by 16 theoretical approaches innovatively applied, based on different algorithms to solve pharmacokinetics matters. The similarities between experimental and calculated values were evaluated using PCA and CA as a statistical tool. The outcomes from statistical analysis imply that among investigated parameters, plasma protein binding is the most complex, and further work in the improvement of in silico approaches in this area is wanted.

## Figures and Tables

**Table 1:** Summary of the lipophilicity and pharmacokinetic properties obtained by theoretical approaches.

Parameter	Curcumin	Demetoxycurcumin	Bisdemetoxycurcumin	α-Boswellic Acid	β-Boswellic Acid	11-Keto-β-boswellic Acid	3-Acetyl-11-keto-β-boswellic Acid	Andrographolide	Neoandrographolide	Piperine
Lipophilicity										
iLOGP	3.27	2.78	1.75	3.99	3.96	3.32	3.80	2.61	3.37	3.38
XLOGP3	3.20	3.32	3.26	8.41	8.26	7.20	7.22	2.16	2.63	3.46
WLOGP	3.15	3.14	3.13	7.23	7.09	6.27	6.84	1.96	1.85	2.51
MLOGP	1.47	1.80	2.13	5.82	5.82	4.87	5.14	1.98	1.26	2.39
Silicos-IT LogP	4.04	3.95	3.87	5.85	5.46	5.15	5.67	2.94	2.55	3.41
SwissADME LogP	3.03	3.00	2.83	6.26	6.12	5.36	5.74	2.33	2.33	3.03
ALOGPs	3.62	3.54	3.46	7.42	6.46	5.50	5.90	1.57	1.88	3.38
miLogP	2.30	2.48	2.67	6.72	6.79	5.69	6.39	1.05	1.17	3.33
Mcule logP	3.37	3.36	3.35	7.23	7.09	6.27	6.84	1.96	1.85	2.94
cLogP	2.95	3.02	3.09	6.06	6.00	5.29	5.78	1.88	1.74	3.60
MolLogP	2.83	2.88	2.93	5.86	5.68	4.66	5.17	2.32	2.51	3.47
ALOGP98	3.22	3.24	3.26	6.42	6.47	5.54	5.92	2.06	2.38	2.86
pkCSM logP	3.37	3.36	3.35	7.23	7.09	6.27	6.84	1.96	1.85	3.00
SKlog_P	3.72	3.49	3.25	6.78	6.61	5.72	6.02	2.10	2.42	3.03
ALOGP	3.37	3.36	3.35	7.23	7.09	6.27	6.84	1.96	1.85	3.00
Solubility										
log *S*_SILICOS-IT_	−4.45	−4.34	−4.23	−6.12	−5.67	−5.55	−6.15	−2.69	−2.70	−3.00
log *S*_ALOGPS_	−4.81	−4.70	−4.46	−5.86	−5.97	−5.51	−6.06	−3.09	−3.37	−3.28
log *S*_AquaSol_	−3.92	−4.01	−4.06	−5.13	−5.20	−4.71	−4.87	−2.78	−3.42	−3.55
log *S*_preADMET_	−4.53	−4.05	−3.56	−7.35	−7.49	−6.92	−7.28	−3.12	−4.44	−3.71
log *S*_admetSAR_	−3.36	−3.09	−2.88	−3.85	−3.79	−3.45	−4.67	−2.85	−3.31	−3.40
Absorption										
HIA_pkCMS_ (%)	82.190	91.393	91.159	94.363	97.466	98.826	99.510	95.357	62.263	94.444
HIA_preADMET_ (%)	94.403	94.029	93.750	95.996	95.996	96.714	99.202	87.688	81.330	98.180
HIA_admetSAR_ (%)	97.70	97.70	97.37	98.53	98.53	99.01	99.19	98.22	81.24	96.39
HOB_admetSAR_ (%)	60.00	64.29	64.29	50.00	52.86	51.43	51.43	61.43	65.71	57.14
Plasma protein binding										
PPB_preADMET_ (%)	88.030	89.913	93.826	100.000	100.000	100.000	94.061	94.873	93.373	90.449
PPB_admetSAR_ (%)	64.0	68.9	42.1	51.4	65.8	62.9	79.4	49.4	52.5	100.0

**Table 2:** Shake-flask method validation data.

Analyte	Linearity Range (µg/mL)	Regression Equation *	*r*	LOD (µg/mL) **	LOQ (µg/mL) ***
Curcumin	1–375	y = 987.3 *x* + 0.935	0.9999	0.3	1
Demetoxycurcumin	1–375	y = 421.0 *x* + 0.610	0.9998	0.3	1
Bisdemetoxycurcumin	10–375	y = 134.4 *x* + 0.200	0.9999	3	10
Andrographolide	5–300	y = 15,039 *x* + 35.28	0.9996	2	5
Neoandrographolide	5–250	y = 1723 *x* + 3.188	0.9995	2	5
Piperine	5–300	y = 10,601 *x* + 10.15	0.9999	2	5
**Analyte**	Precision (RSD, %) ****		Accuracy (Recovery and RSD, %) *****		
	**Repeatability** **(*n* = 6)**	**Intermediate Precision (*n* = 9)**	**Low** **(*n* = 3)**	**Medium** **(*n* = 3)**	**High** **(*n* = 3)**
Curcumin	0.42	1.59	102.5 ± 3.89	95.0 ± 3.55	97.5 ± 2.95
Demetoxycurcumin	0.38	0.80	102.9 ± 3.81	95.4 ± 3.41	98.8 ± 3.01
Bisdemetoxycurcumin	0.98	0.27	103.0 ± 3.40	96.2 ± 3.31	99.4 ± 2.55
Andrographolide	1.61	3.61	97.2 ± 3.66	99.1 ± 1.98	98.4 ± 1.59
Neoandrographolide	1.73	3.39	98.1 ± 2.90	102.1 ± 2.50	96.5 ± 2.34
Piperine	0.73	0.59	102.5 ± 0.92	104.3 ± 0.94	103.0 ± 0.91

* Linearity was examined on at least five concentration levels in three individual standard solution preparations from which a single regression line was constructed. ** Limit of Detection (LOD) was determined using signal-to-noise value 3. *** Limit of Quantitation (LOQ) was determined using signal-to-noise value 10. **** Repeatability was assessed analysing six individual samples on the same day, while intermediate precision was examined on three individual samples over three days. Results are expressed as Relative Standard Deviations (RSD). ***** Accuracy of the method was examined by analysis of standard solutions in triplicate on three concentration levels. Results are expressed both as recoveries and Relative Standard Deviations (RSD).

**Table 3:** Log D_7.4_ values * of biologically active ingredients of botanicals used in IBD treatment determined by applied shake-flask procedure.

Analyte	log *D*_7.4_ ± RSD ** (%) (*n* = 3)
Curcumin	1.94 ± 2.59
Demetoxycurcumin	1.52 ± 3.78
Bisdemetoxycurcumin	1.72 ± 4.21
Andrographolide	1.32 ± 3.25
Neoandrographolide	1.51 ± 2.55
Piperine	1.67 ± 3.55

* Log *D*_7.4_—the logarithm of the distribution coefficient between *n*-octanol and buffer phase; describes the distribution of all forms of the compound at a specific pH. ** RSD—Relative Standard Deviation.

**Table 4:** Hydrophobicity and lipophilicity parameters determined by chromatographic assays.

Analyte	Linear Equation *	*r*	Standard Error
C18-TLC Assay			
Curcumin	y = −0.0540 x + 4.4855	0.9916	0.0835
Demetoxycurcumin	y = −0.0504 x + 4.1781	0.9932	0.0699
Bisdemetoxycurcumin	y = −0.0503 x + 4.1751	0.9937	0.0675
11-keto-β-boswellic acid	y = −0.0564 x + 5.3756	0.9950	0.0399
3-acetyl-11-keto-β-boswellic acid	y = −0.0722 x + 6.9059	0.9798	0.1041
Andrographolide	y = −0.0302 x + 2.0908	0.9970	0.0277
Neoandrographolide	y = −0.0334 x + 2.1672	0.9964	0.0266
Piperine	y = −0.0526 x + 4.4044	0.9789	0.0913
C18-HPLC Assay			
Curcumin	y = −0.0442 x + 3.4187	0.9918	0.0368
Demetoxycurcumin	y = −0.0448 x + 3.4349	0.9827	0.0542
Bisdemetoxycurcumin	y = −0.0447 x + 3.4174	0.9818	0.0556
Andrographolide	y = −0.0271 x + 1.9065	0.9695	0.0581
Neoandrographolide	y = −0.0359 x + 2.8595	0.9782	0.0489
Piperine	y = −0.0394 x + 3.4218	0.9796	0.0519
IAM-HPLC Assay			
Curcumin	y = −0.0565 x + 4.3463	0.9969	0.0530
Demetoxycurcumin	y = −0.0555 x + 4.4229	0.9941	0.0554
Bisdemetoxycurcumin	y = −0.0568 x + 4.6386	0.9940	0.0569
α-boswellic acid	y = −0.0788 x + 6.3109	0.9956	0.0526
β-boswellic acid	y = −0.0816 x + 6.5157	0.9982	0.0346
11-keto-β-boswellic acid	y = −0.0570 x + 4.9194	0.9874	0.0645
3-acetyl-11-keto-β-boswellic acid	y = −0.0703 x + 5.1157	0.9944	0.0526
Andrographolide	y = −0.0464 x + 2.2763	0.9796	0.0753
Neoandrographolide	y = −0.0620 x + 3.4572	0.9854	0.0755
Piperine	y = −0.0512 x + 3.2026	0.9830	0.0872

* Depending on the applied methodology, the intercept of linear regression represents hydrophobicity/lipophilicity indices *R*_M0_, log*k*_0_ and log*k*_0 IAM_, respectively. The indices were derived by extrapolation using the following equations: *R*_M_ = *R*_M0_—S x φ for C18-TLC assay, log*k* = log*k*_0_—S x φ for C18-HPLC assay, and log*k* _IAM_ = log*k*_0 IAM_—S x φ for IAM-HPLC assay. φ represents the volume fraction of the organic modifier in the mobile phase and S a constant derived by linear regression analysis.

**Table 5:** Plasma protein binding data (%) determined by HSA-HPAC and AGP-HPAC assays.

Analyte	HSA Binding ± RSD * (%) (*n* = 3)	AGP Binding ± RSD (%) (*n* = 3)
Curcumin	97.52 ± 0.20	83.07 ± 1.10
Demetoxycurcumin	97.97 ± 0.35	82.92 ± 1.15
Bisdemetoxycurcumin	98.37 ± 0.10	83.14 ± 1.15
α-boswellic acid	98.94 ± 0.35	89.22 ± 0.30
β-boswellic acid	99.79 ± 0.30	89.15 ± 2.04
11-keto-β-boswellic acid	98.09 ± 0.02	79.01 ± 0.20
3-acetyl-11-keto-β-boswellic acid	99.83 ± 0.47	83.10 ± 0.37
Andrographolide	80.07 ± 0.74	37.13 ± 1.11
Neoandrographolide	87.71 ± 0.41	64.95 ± 0.41
Piperine	95.48 ± 0.41	71.98 ± 1.33

* RSD—Relative Standard Deviation.

## Data Availability

Data are contained within the article and supplementary materials.

## Supplementary Materials

The following supporting information can be downloaded at: https://www.mdpi.com/article/10.3390/ph15080965/s1, Table S1. Verification data of HSA-HPAC and AGP-HPAC assays.

## References

[1] Jairath V., Feagan B.G. (2020). Global burden of inflammatory bowel disease. Lancet Gastroenterol. Hepatol..

[2] Lin S.C., Cheifetz A.S. (2018). The use of complementary and alternative medicine in patients with inflammatory bowel disease. Gastroenterol. Hepatol..

[3] Governa P., Marchi M., Cocetta V., De Leo B., Saunders P.T.K., Catanzaro D., Miraldi E., Montopoli M., Biagi M. (2018). Effects of Boswellia Serrata Roxb. and Curcuma longa L. in an in vitro intestinal inflammation model using immune cells and Caco-2. Pharmaceuticals.

[4] Mishra A., Shaik H.A., Sinha R.K., Shah B.R. (2021). Andrographolide: A herbal-chemosynthetic approach for enhancing immunity, combating viral infections, and its implication on human health. Molecules.

[5] Coelho M.R., Romi M.D., Ferreira D.M.T.P., Zaltman C., Soares-Mota M. (2020). The use of curcumin as a complementary therapy in ulcerative colitis: A systematic review of randomized controlled clinical trials. Nutrients.

[6] Algieri F., Rodriguez-Nogales A., Rodriguez-Cabezas M.E., Risco S., Angeles Ocete M., Galvez J. (2015). Botanical drugs as an emerging strategy in inflammatory bowel disease: A review. Mediat. Inflamm..

[7] Li Q., Zhai W., Jiang Q., Huang R., Liu L., Dai J., Gong W., Du S., Wu Q. (2015). Curcumin-piperine mixtures in self-microemulsifying drug delivery system for ulcerative colitis therapy. Int. J. Pharm..

[8] Ermondi G., Vallaro M., Caron G. (2018). Learning how to use IAM chromatography for predicting permeability. Eur. J. Pharm. Sci..

[9] Carrasco-Correa E.J., Ruiz-Allica J., Rodríguez-Fernández J.F., Miró M. (2021). Human artificial membranes in (bio)analytical science: Potential for in vitro prediction of intestinal absorption-A review, *TrAC-Trend*. Anal. Chem..

[10] Obradović D., Radan M., Đikić T., Popović Nikolić M., Oljačić S., Nikolić K. (2022). The evaluation of drug-plasma protein binding interaction on immobilized human serum albumin stationary phase, aided by different computational approaches. J. Pharmaceut. Biomed. Anal..

[11] Jeličić M.-L., Brusač E., Klarić D.A., Nigović B., Turk N., Mornar A. (2020). A chromatographic approach to development of 5-aminosalicylate/folic acid fixed-dose combinations for treatment of Crohn’s disease and ulcerative colitis. Sci. Rep..

[12] Brusač E., Jeličić M.-L., Amidžić Klarić D., Nigović B., Turk N., Klarić I., Mornar A. (2019). Pharmacokinetic profiling and simultaneous determination of thiopurine immunosuppressants and folic acid by chromatographic methods. Molecules.

[13] Russo G., Grumetto L., Baert M., Lynen F. (2021). Comprehensive two-dimensional liquid chromatography as a biomimetic screening platform for pharmacokinetic profiling of compound libraries in early drug development. Anal. Chim. Acta.

[14] Abdel-Tawab M. (2021). Considerations to be taken when carrying out medicinal plant research—what we learn from an insight into the IC50 values, bioavailability and clinical efficacy of exemplary anti-inflammatory herbal components. Pharmaceuticals.

[15] Sharma T., Jana S. (2020). Investigation of molecular properties that influence the permeability and oral bioavailability of major β-boswellic acids. Eur. J. Drug Metab. Pharmacokinet..

[16] Krüger P., Daneshfar R., Eckert G.P., Klein J., Volmer D.A., Bahr U., Müller W.E., Karas M., Schubert-Zsilavecz M., Abdel-Tawab M. (2008). Metabolism of boswellic acids in vitro and in vivo. Drug Metab. Dispos..

[17] Godugu D., Rupula K., Sashidhar R.B. (2018). Binding studies of andrographolide with human serum albumin: Molecular docking, chromatographic and spectroscopic studies. Protein Pept. Lett..

[18] Kempińska D., Chmiel T., Kot-Wasik A., Mróz A., Mazerska Z., Namieśnik J. (2019). State of the art and prospects of methods for determination of lipophilicity of chemical compounds. TrAC-Trend. Anal. Chem..

[19] Brusač E., Jeličić M.-L., Amidžić Klarić D., Mornar A. (2019). Miniaturized shake-flask HPLC method for determination of distribution coefficient of drugs used in inflammatory bowel diseases. Acta Pharm..

[20] International Conference on Harmonization of Technical Requirements for Registration of Pharmaceuticals for Human Use, ICH Harmonised Tripartite Guideline. Validation of Analytical Procedures: Text and Methodology Q2(R1), Current Step 4 Version. November 2005. https://www.ich.org/fileadmin/Public_Web_Site/ICH_Products/Guidelines/Quality/Q2_R1/Step4/Q2_R1__Guideline.pdf.

[21] Kotagale N.R., Charde P.B., Helonde A., Gupta K.R., Umekar M.J., Raut N.S. (2020). Studies on bioavailability enhancement of curcumin. Int. J. Pharm. Pharm. Sci..

[22] Jithavech P., Suwattananuruk P., Muangnoi C., Thitikornpong W., Towiwat P., Vajragupta O., Rojsitthisak P. (2022). Physicochemical investigation of a novel curcumin diethyl γ-aminobutyrate, a carbamate ester prodrug of curcumin with enhanced antineuroinflammatory activity. PLoS ONE.

[23] Roy N.K., Parama D., Banik K., Bordoloi D., Devi A.K., Thakur K.K., Padmavathi G., Shakibaei M., Fan L., Sethi G. (2019). An Update on pharmacological potential of boswellic acids against chronic diseases. Int. J. Mol. Sci..

[24] Dei Cas M., Ghidoni R. (2019). Dietary curcumin: Correlation between bioavailability and health potential. Nutrients.

[25] Boyle C.A., Coatney R.W., Wickham A., Mukherjee S.K., Meunier L.D. (2021). Alpha-1 acid glycoprotein as a biomarker for subclinical illness and altered drug binding in rats. Comp. Med..

[26] Dezhampanah H., Shabanzade Z. (2022). Investigation of binding interaction between human serum albumin with zirconium complex of curcumin and curcumin. J. Biomol. Struct. Dyn..

