# Supplementary material for: Accessing Lipophilicity and Biomimetic Chromatography Profile of Biologically Active Ingredients of Botanicals Used in the Treatment of Inflammatory Bowel Disease
Source: Pharmaceuticals (Basel). 2022 Aug 4;15(8):965. doi: 10.3390/ph15080965 (PMC9413514; doi:10.3390/ph15080965)
Supplement: Supplementary file 1 [file pharmaceuticals-15-00965-s001.zip › pharmaceuticals-1835683-supplementary.pdf]

SUPPLEMENTARY MATERIAL

Accessing Lipophilicity and Biomimetic Chromatography Profile of Biologically Active Ingredients of Botanicals Used in the Treatment of Inflammatory Bowel Disease

Mario-Livio Jeličić, Daniela Amidžić Klarić, Jelena Kovačić, Donatella Verbanac, Ana Mornar

Table S1. Verification data of HSA-HPAC and AGP-HPAC assays.

Assay	t_{R1}* (min) ± RSD** (%) n = 6	t_{R2} (min) ± RSD (%) n = 6	R_s***± RSD (%) n = 6
**HSA-HPAC**	6.03 ± 0.56	6.69 ± 0.56	2.27 ± 1.93
**AGP-HPAC**	4.05 ± 0.54	4.54 ± 0.36	1.63 ± 1.04

*t_R – retention time of peaks in the racemic mixture of warfarin

**RSD – Relative Standard Deviation

***R_s – resolution factor
